# Assessment of the Additive Effect of Remineralizing Agents in Combination With Fluoride Releasing Adhesives in the Prevention of Enamel Decalcification in Orthodontic Patients: An In Vitro Study

**DOI:** 10.7759/cureus.62337

**Published:** 2024-06-13

**Authors:** Indira Senthilkumar, Perala Johnson, Siva Krishna Polisetty, Gowri Sankar Singaraju, Ganugapanta Vivek Reddy, Prasad Mandava

**Affiliations:** 1 Orthodontics and Dentofacial Orthopaedics, Narayana Dental College, Nellore, IND; 2 Orthodontics and Dentofacial Orthopaedics, Government Dental College and Hospital, Kadapa, IND

**Keywords:** varnish, remineralization, orthodontic, hardness, fluoride, enamel, cpp-acp, bond strength, bracket, adhesive

## Abstract

Introduction

Incorporation of remineralizing agents with fluoride-releasing bracket adhesives may prevent the development of white spot lesions (WSL) or reverse the established WSL in patients undergoing fixed orthodontic treatment. We aimed to find out how effectively casein phosphopeptide-amorphous calcium phosphate (CPP-ACP) and fluoride varnish (FV) can remineralize teeth when mixed with fluoride-releasing orthodontic adhesive.

Materials and methods

We randomly assigned a total of 60 premolar teeth, therapeutically extracted for orthodontic purposes, into two equal groups. Group I (n = 30) utilized fluoride-releasing adhesive (FR), and Group II (n = 30) bonded with non-fluoride adhesive (NFR). Based on the applied remineralizing agent, we further divided each of the two groups into three equal subgroups of 10: Group IA (FR+FV), Group IB (FR+CPP-ACP), Group IC (control-only FR), Group IIA (NFR+FV), Group IIB (NFR+CPP-ACP), and Group IIC (control-only NFR). Following bonding procedures, all the samples underwent pH cycling for 28 days, where the enamel samples were immersed in 20 ml of demineralizing solution for three hours, followed by immersion in 30 ml of remineralizing solution for 17 hours. The samples were analyzed for shear bond strength (SBS) on a universal testing machine and hardness values (HV) by the Vickers microhardness test (VMT) using the indentation method. We also evaluated the adhesive remnant index (ARI) scores to determine the site of bracket failure.

Statistical analysis

The shear bond strength (SBS) and hardness value (HV) were expressed as the mean, standard deviation (SD), and median for each subgroup. We used the non-parametric Kruskal-Wallis test to analyze the SBS and HV, followed by the Dunn-Bonferroni test for intra-pair differences. The ARI score was expressed as the frequency of the percentage distribution, and the difference in the distribution of ARI scores between the groups was assessed by the Cochran chi-square test. The probability (p) value equal to or less than 0.05 was considered statistically significant.

Results

The results show that Group IB, bonded with a fluoride-releasing adhesive and a CPP-ACP remineralizing agent surface treatment, has the highest HV of 300.23 units. Group IIC (only NFR) has the lowest hardness of 153.3 units, which is statistically significant (p < 0.001). However, the ARI scores are not statistically significant between the groups tested.

Conclusion

The bond strength of the adhesive and the surface hardness of the enamel increased with the addition of fluoride varnish and CPP-ACP to both the fluoride-releasing and non-fluoride-releasing adhesives.

## Introduction

Demineralization of enamel is a common problem for both orthodontists and patients undergoing fixed orthodontic treatment. This phenomenon arises because of the accumulation of plaque, which contains acidogenic bacteria, in close proximity to the brackets. The acid released by this bacteria creates an environment that is conducive to demineralization [1]. White spot lesions (WSL) serve as the primary clinical indicator of dental caries and represent the initial stage of enamel demineralization [2]. Enamel demineralization can be reversed if diagnosed at the earliest possible time and treated promptly [3]. Studies have demonstrated that between 50 and 97% of individuals who received fixed orthodontic appliances within a month developed WSL [4].

A multifactorial approach is needed to effectively combat the initiation and progression of WSL, and one such product is remineralizing agents. Remineralizing compounds control the demineralization/remineralization cycle in favor of enamel. These compounds work by influencing the microenvironment surrounding the tooth [5]. Remineralizing agents, such as fluoride varnish, self-assembling peptide, sodium phosphosilicate, sugar substitutes, calcium phosphate systems, ozone, hydroxyapatite, and resin infiltrates, are available on the market to aid in the process of remineralizing the tooth structure [6]. Calcium phosphate systems include casein-phosphopeptide-amorphous calcium fluoride phosphate (CPP-ACFP) and casein-phosphopeptide-amorphous calcium phosphate (CPP-ACP) [6].

Fluoride is one of the key ingredients in these agents that might help avoid tooth decay and comes in a variety of forms. Fluoride varnish, a product with a 5% concentration, is a popular choice among patients and dentists due to its ease of application, minimal ingestion risk, and patient approval [7]. Another non-invasive method for preventing enamel demineralization is to replace calcium, phosphate, and fluoride ions in the enamel, which increases its strength [8]. Casein phosphopeptide-amorphous calcium phosphate (CPP-ACP) is one such agent based on casein, a bioactive product derived from milk. Calcium-phosphate-based systems improve the available calcium and phosphate in the oral cavity. These are supplied in paste form and are commercially available as tooth mousse [9].

The concept of developing orthodontic composites with fluoride-releasing properties emerged to prevent WSL around the bracket without relying on patient cooperation. Orthodontic treatment commonly uses composite resins and glass ionomers as adhesive groups [2]. Though glass ionomers are capable of releasing more fluoride than composite resin, this material exhibits less bond strength. However, the levels of fluorides released from the composites are too low to effectively combat the development of white spot lesions [2]. Hence, it is important to find a better way of delivering fluorides, and it is prudent to mix the remineralizing agent with the fluoride-releasing orthodontic adhesive composite to enhance the additive effect of the remineralizing capacity of the fluoride around the orthodontic brackets.

The current study aims to assess the effectiveness of these two remineralizing agents when combined with fluoride and non-fluoride-releasing orthodontic adhesives by artificially inducing demineralizing lesions on the extracted premolar teeth. 

## Materials and methods

This in vitro experiment conducted as part of a thesis procedure was evaluated and authorized by the Institutional Ethical Committee at Narayana Dental College, Nellore, India. The approval number for this investigation is IEC/NDCH/2022/Mar/P.-25, dated March 28, 2022.

Based on a power analysis using data from prior research [1,6,8], it was determined that a sample size of 48 specimens would provide 80% power to detect a difference of 10 in surface hardness (effect size of 0.4) between the mean levels of any two groups, with a 0.05 level of error (G*Power version 3.1.9.6). Nevertheless, the study utilized a sample size of 60 individuals.

A total of 60 healthy human premolars were chosen for this investigation, specifically extracted for orthodontic treatment purposes. Teeth with cavities, fillings, endodontic treatment, enamel hypoplasia, and enamel cracks were deliberately excluded from the study. The extracted teeth were preserved in a solution containing 0.1% thymol.

**Figure 1:**
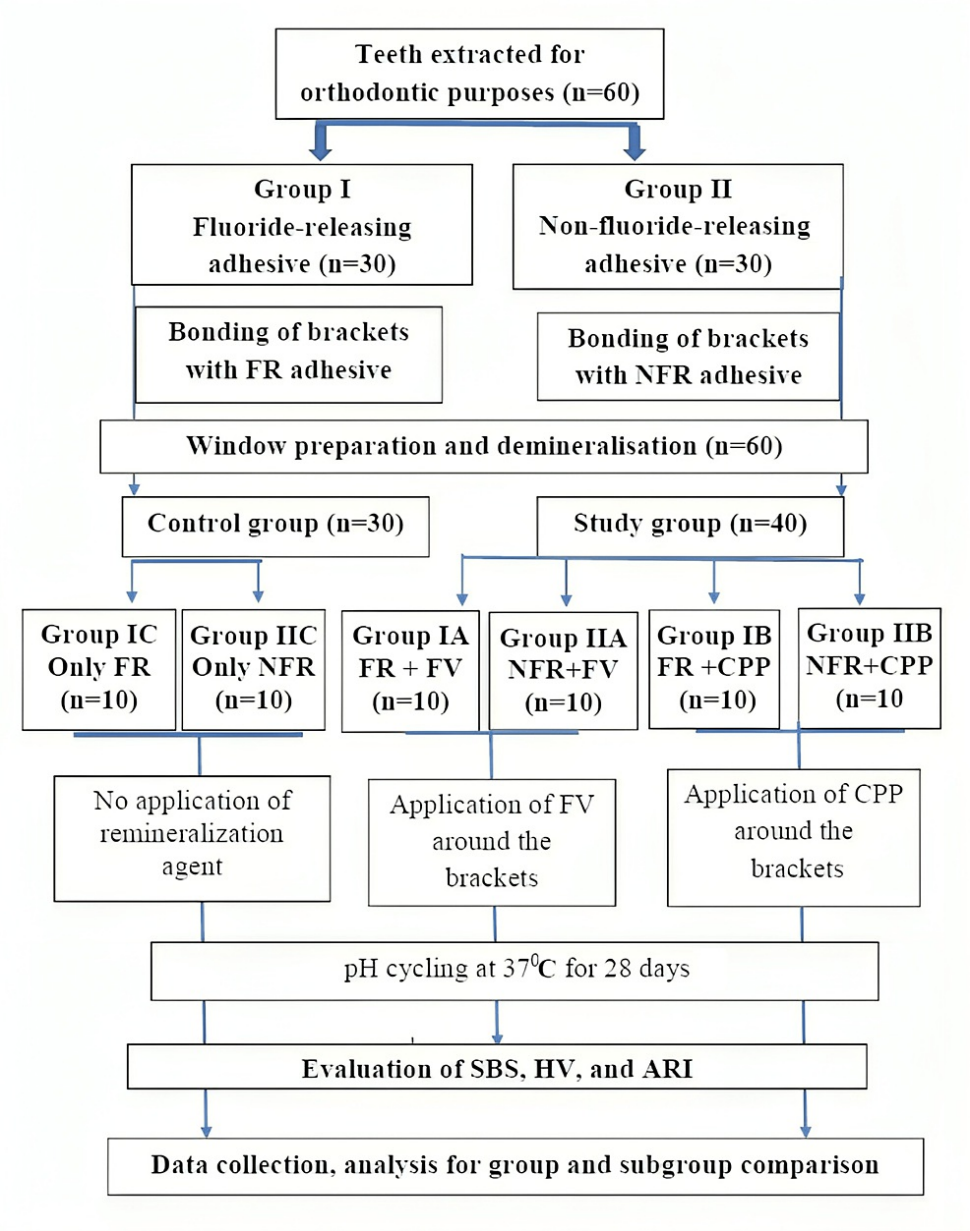
Flow chart showing the methodology of the study. GI-C (FR): only fluoride-releasing adhesive; GII-C (NFR): only non-fluoride-releasing adhesive; GI-A (FR+FV): fluoride-releasing+fluoride varnish; GI-B (FR+CPP): fluoride-releasing+CPP-ACP; GII-A (NFR+FV): non-fluoride-releasing+fluoride varnish; G II-B (NFR+CPP): non-fluoride-releasing+CPP-ACP; SBS: shear bond strength; HV: hardness value; ARI: adhesive remnant index; CPP-ACP: casein phosphopeptide-amorphous calcium phosphate

**Figure 2:**
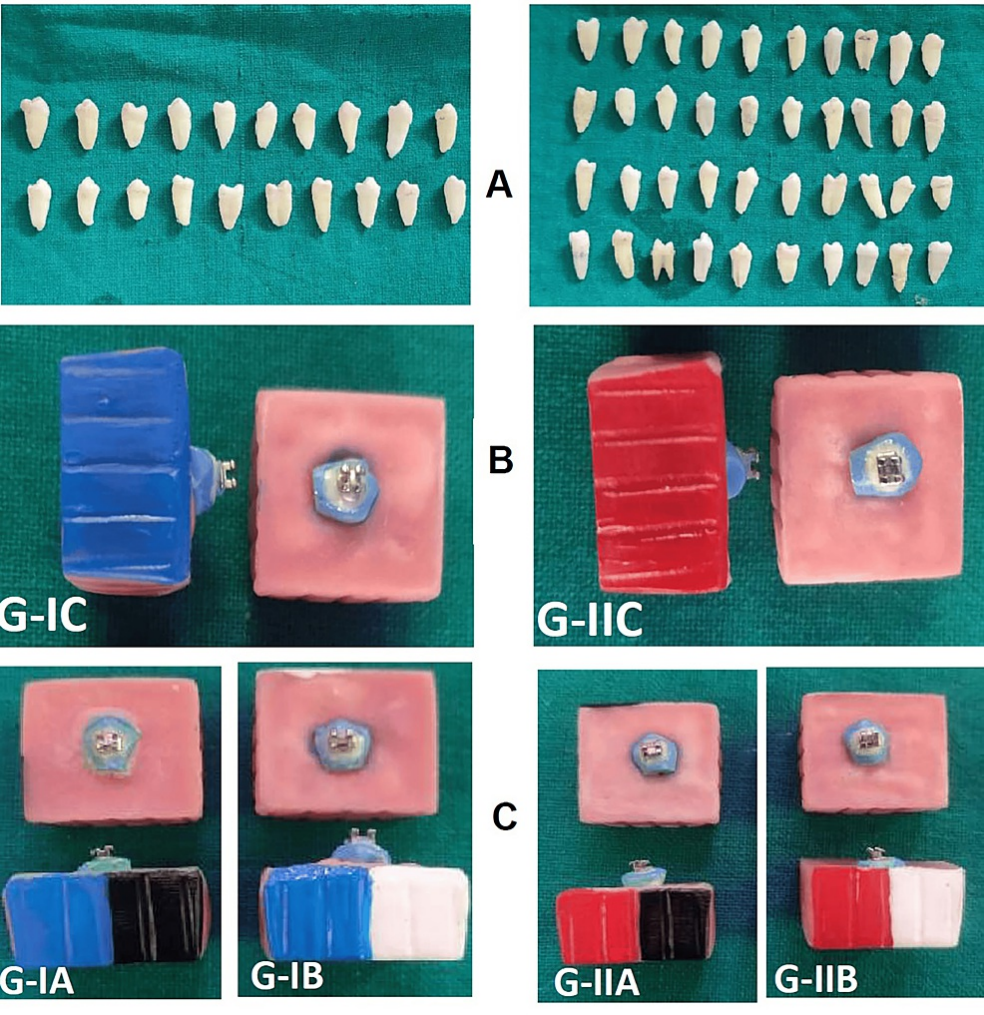
Sample distribution of the control groups and the test groups. A: extracted premolar teeth; B: color coding of the acrylic blocks-control groups, Group IC (blue) is fluoride-releasing adhesive (FR), and Group IIC (pink) is non-fluoride-releasing (NFR); C: color coding of the acrylic blocks, test Group IA (blue-black) is FR+FV, Group IB (blue-white) is FR+CPP, Group IIA (pink-black) is NFR+FV, and Group IIB (pink-white) is NFR+CPP. FV: fluoride varnish; CPP: casein phosphopeptide; NFR: non-fluoride adhesive

This study employed two distinct orthodontic adhesive systems, Group I fluoride-releasing adhesive (FR) and Group II non-fluoride-releasing adhesive (NFR), with a sample size of n = 30 for each group. We divided each primary group into three equal subgroups of ten samples each (n = 10). The remineralizing treatment subgroups utilized either fluoride varnish (FV) or casein-phosphopeptide-amorphous calcium phosphate (CPP-ACP). Thus, we had Group IA (FR+FV), Group IB (FR+CPP-ACP), Group IC (control-only FR), Group IIA (NFR+FV), Group IIB (NFR+CPP-ACP), and Group IIC (control-only NFR). There is no remineralizing agent in either of the control subgroups. (Figure 1, 2).

We conducted the procedure in two phases, with some sequential steps. In the first phase, we bonded all the sample teeth with orthodontic adhesives, and in the second phase, we coated the orthodontic adhesive with the remineralizing agent in the test groups.

Phase I

Step 1: Slicing and Mounting

**Figure 3:**
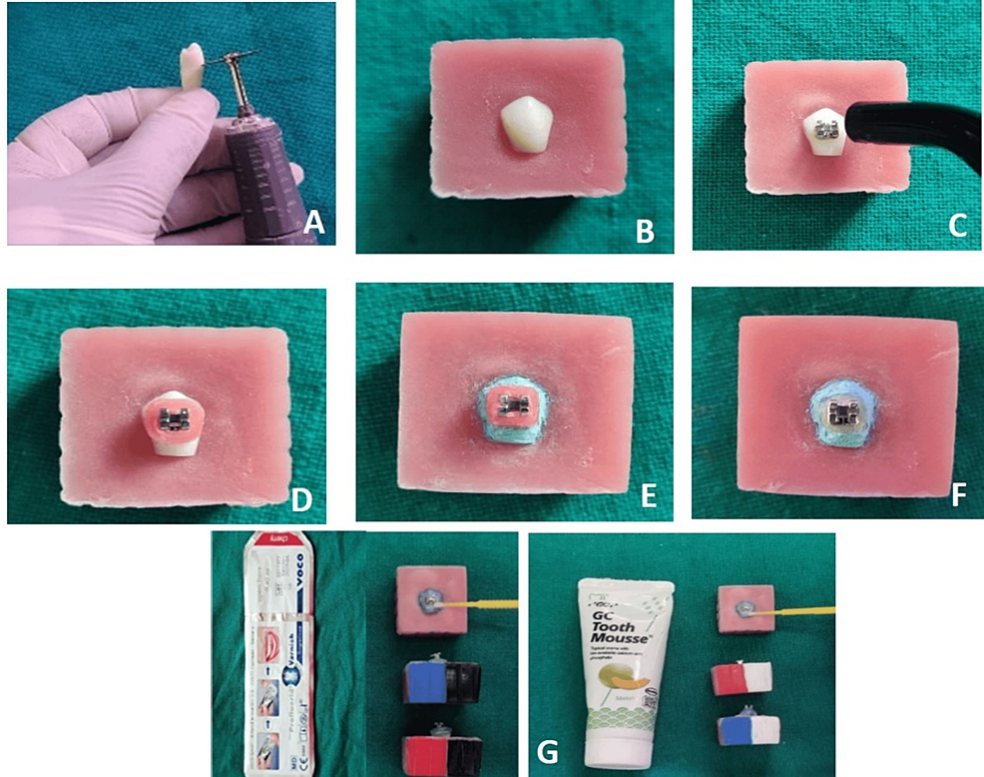
Procedural steps. A: sample slicing; B: acrylization of the sliced sample; C: bonding of brackets; D: window preparation; E: nail varnish application; F: post-demineralization; G: application of remineralizing agent to the demineralized surface

After collection, we sectioned the samples horizontally at the cervical region near the cementoenamel junction using a 22-mm diamond cutting disc (Deccan Dental Depot Pvt. Ltd.), dividing the tooth into two halves: the crown and the root. We then sectioned the crown portion of the tooth mesiodistally, forming the buccal and lingual segments, and selected the buccal segment for the study. After slicing the samples, we embedded them in a 25x25mm acrylic block using the mould (Figure 3).

Step 2: Sample Allocation and Bracket Bonding

We randomly divided the samples into two main groups (n = 30). We coded Group I as blue and Group II as pink. After that, we etched all the samples for 15 seconds each with 37% phosphoric acid (Smart Etch, Safe Endo) and rinsed them with running water. We dried the enamel samples using an oil-free, three-way syringe and ensured they had a frosty white appearance. In all groups, we used stainless steel brackets (3.6mm×2.4mm) (Ormco, Mini Diamond). The FR adhesive system, Trandbond XT primer, and Transbond Plus adhesive (3M Unitek) bonded the Group I brackets. The Group II samples were bonded by the NFR adhesive system: Trandbond XT primer and Transbond XT (3M Unitek). The brackets were light-cured (Woodpecker light-cure LED.D., Guilin Woodpecker Medical Instruments Co., Ltd., China) for 10 seconds at a light intensity of 680 mW/cm2 (Figure 2, 3). 

Step 3: Window Preparation

After bonding the samples, a wax sheet (Hindustan, India) of 6mm×5mm size was adapted on and around the bracket, ensuring that at least 1 mm of tooth surface was covered in all directions around the periphery of the bracket, which was placed in the center. We coated the remaining tooth surface with nail varnish to make it resistant to the demineralization procedures in the subsequent steps. We removed the wax from the teeth's surface after they had been dried, exposing the window portion to demineralization procedures around the bracket (Figure 3).

Step 4: Demineralization of the Sample

We simulated the induction of artificial caries around the bracket borders by placing all the samples in the demineralizing solution containing acetate 0.1 mol/L, calcium 0.1 mol/L, phosphate 0.1 mol/L, fluoride 0.1 mg/L, and pH 5.0.The samples were kept in an incubator at a temperature of 37°C for approximately four days (Figure 3).

Step 5: pH Cycling

Ten samples from each main group that serve as controls (Group IC and Group IIC) were subjected to pH cycling for 28 days. This process replicated the conditions of the oral cavity in a laboratory setting, where demineralization and remineralization occur in alternating cycles. Initially, we immersed the samples in 20 ml of demineralizing solution (calcium 2.0 mmol/L, phosphate 2.0 mmol/L, acetic acid 75.0 mmol/L, pH 4.4) for three hours. This is followed by immersion of the enamel samples in 30 ml of remineralizing solution (1.5 mmol calcium, 0.9 mmol phosphate, 0.15 mol Kcl in 0.1 m Tris buffer, pH 7) for 17 hours. We replaced the remineralizing solution every 48 hours and the demineralizing agent every five days. We carried out the pH cycling for 28 days [1].

Step 6: Evaluation of the SBS, HV, and ARI Scores of the Control Groups

**Figure 4:**
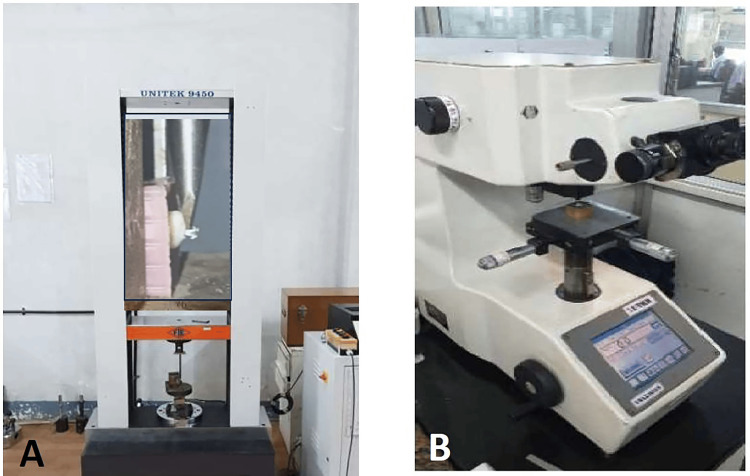
Equipment used for assessing shear bond strength (SBS) and hardness values (HV). A: Universal testing machine (50kn/2.5kn, Make: FIE, Model: UNITEK 9450) with inset showing shear bond strength testing on the sample; B: Vickers microhardness tester (Digital microhardness tester, Matsuzawa Co., Ltd., Model: MMT X7, Japan)

**Figure 5:**
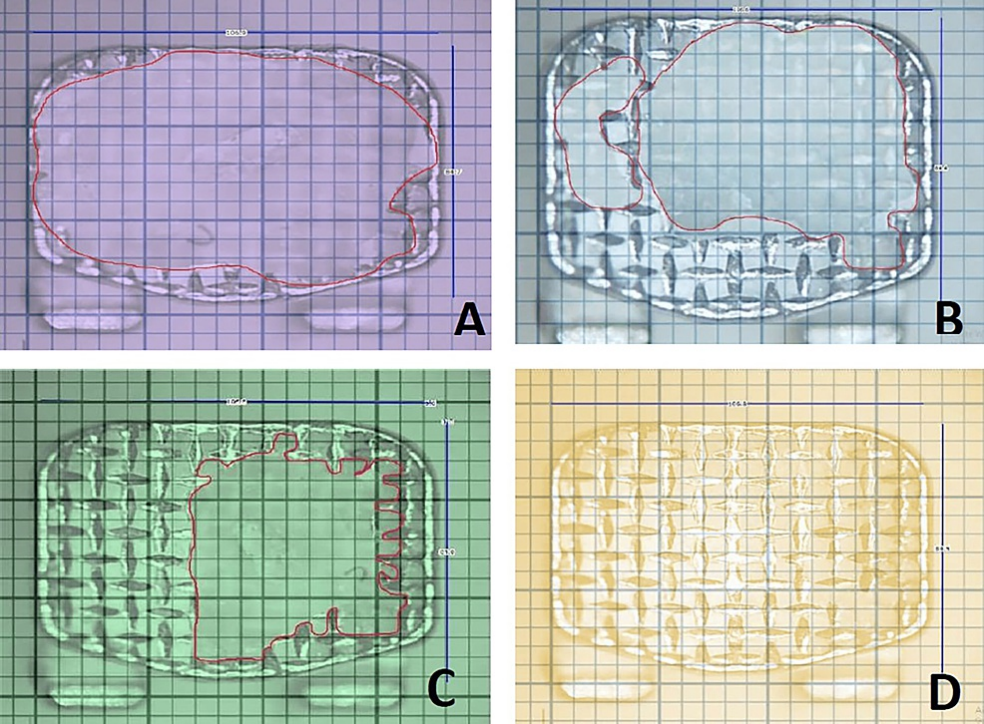
ARI scores. A: score 0-bracket base with 100% adhesive; B: score 1-bracket base with more than 50% adhesive; C: score 2-bracket base with less than 50% adhesive; D: score 3-bracket base with no adhesive

After 28 days of pH cycling, we evaluated the shear bond strength (SBS) of the control samples (Group IC and Group IIC) using a universal testing machine. The digital microhardness tester employed the VMT to assess the surface hardness of the enamel. Following the SBS measurement, one of the investigator (IPS) assessed the ARI scores by inspecting the bases of the brackets that became debonded during the procedure (Figure 4, 5).

Phase II 

The remaining samples (n = 20) in each of the two main groups (FR and NFR) were divided into two subgroups based on the application of the remineralization agents FV and CPP-ACP. Thus, we have four test subgroups: Group IA (FR+FV), Group IB (FR+CPP-ACP), Group IIA (NFR+FV), and Group IIB (NFR+CPP-ACP). 

Step 7: Application of Fluoride Varnish and CPP-ACP

The acrylic blocks of the fluoride varnish (FV) samples (Group IA and Group IIA) were coated black, while the CPP-ACP groups (Group IIA and Group IIB) were painted white on one of the sides of the main acrylic blocks. The samples in each group, according to their allocation, were subjected to treatment with either a layer of fluoride varnish containing 5% sodium fluoride, 22,600 ppm fluoride (Profluorid Varnish, Voco), or CPP-ACP (GC Tooth Mousse, GC Corp., Tokyo, Japan) applied on and around the surface of the bracket for 15 seconds. We allowed the samples to air dry for one minute after the application.

In Phase II, the ensuing procedures were conducted using the identical techniques as in Phase I for pH cycling. The only difference was that the samples were coated with the representative remineralizing agent each day for 28 days. Subsequently, we assessed the physical characteristics of each of the samples.

Methods of measurement of SBS, HV, and ARI scores

Shear Bond Strength (SBS)

The universal testing machine (Make: FIE, Model: UNITEK 9450, India) has an inbuilt mechanism with a digital monitor to display the debonding forces. The samples were stabilized by mounting them on the lower jaw of the unit, which was equipped with a 50-kg load cell. A knife-edged blade of 0.5 mm edge thickness at a crosshead speed of one millimeter per minute was applied directly parallel to the external surface of the tooth. The point of application of force is directed to the top of each bracket base, specifically at the contact point between the tooth and composite interface. The force at which the specimen debonded was the shear force at fracture and can be directly read on the monitor.

Hardness Values (HV)

The surface microhardness was measured using a Vickers microhardness tester (Digital microhardness tester MMTX7, Matsuzawa, Japan). The test was performed with a quadrangular pyramid-shaped indenter and a constant load of 100 grams for 10 seconds. We made three indentations on the mesial, distal, and upper surfaces around the debonded bracket and recorded the average of these measurements as hardness value units (HV = gm/mm2).

Adhesive Remanent Index (ARI)

The scores were determined by examining the bracket base after debonding using a stereomicroscope (SZ-40 Olympus, Japan) at a magnification of 20X. A calibrated graph paper was overlayed on the microscopic images obtained using Dolphin imaging software (Version 11.9). The bracket base was overlaid with a total of 205 squares. The number of individual squares covered with composite was counted for each bracket, and the percentage of the surface area covered by the composite was derived. The ARI was measured for each bracket in the individual group. The ARI of the tooth surface was derived by applying the unmodified ARI developed by Årtun and Bergland [10].

Statistical analysis

All the continuous quantitative data for the shear bond test (MPa) and hardness value units (HV=gm/mm2) was obtained and entered in an Excel (Microsoft Corporation, Redmond, Washington, United States) sheet. The ARI score was expressed as the frequency of the percentage distribution. The difference between the groups for continuous data was analyzed by the non-parametric Kruskal-Wallis test, followed by the post-hoc Dunn-Bonferroni test to evaluate the intrapair differences. The Cochran chi-square test assesses the difference in frequency of the distribution of ARI scores between groups. The probability (p) value for statistical significance was 0.05 or less for all the analytical tests.

## Results

The descriptive data and comparison between the groups for SBS and HV can be found in Table 1, 2. The continuous variable values among all the groups are compared using the Kruskal-Wallis test, followed by a post hoc test to analyze the differences between each pair of groups (Table 3, 4). The frequency distribution of ARI scores in the individual groups is shown in Table 5. A comparative analysis of the frequency distribution of ARI scores across the groups under study is shown in Table 6.

**Table 1:** Comparison of the shear bond strength (SBS) between groups (Kruskal-Wallis test). Test statistic: Kruskal-Wallis test; * p<0.05 statistically significant. RM: remineralizing agent; GI-C (FR): only fluoride-releasing adhesive; GII-C (NFR): only non-fluoride-releasing adhesive; GI-A (FR+FV): fluoride-releasing+fluoride varnish; GI-B (FR+CPP): fluoride-releasing+CPP-ACP; GII-A (NFR+FV): non-fluoride-releasing+fluoride varnish; GII-B (NFR+CPP): non-fluoride-releasing+CPP-ACP; CPP-ACP: casein phosphopeptide-amorphous calcium phosphate

Group (n = 10) each		Megapascal (MPa)					Mean rank	Test statistic
		Mean	SD	Min	Max	Median		
Control groups (adhesive only)	GI-C (FR)	7.97	0.14	7.7	8.2	8	15.85	χ2=54.85; df=5 ; p=<.001
	GII-C (NFR)	7.56	0.31	6.9	8	7.55	6.7	
Test groups (adhesive plus RM agent)	GI-A (FR+FV)	11.59	0.5	10.5	12.2	11.7	54.45	
	GI-B (FR+CPP)	8.4	0.35	7.9	9	8.4	24.35	
	GII-A (NFR+FV)	9.36	0.4	8.8	10	9.4	35.15	
	GII-B (NFR+CPP)	10.73	0.42	10	11.3	10.75	46.5	

**Table 2:** Comparison of hardness values (HV) between groups (Kruskal Wallis test). Test statistic: Kruskal-Wallis test; * p<0.05 statistically significant. RM: remineralizing agent; GI-C (FR): only fluoride-releasing adhesive; GII-C (NFR): only non-fluoride-releasing adhesive; GI-A (FR+FV): fluoride-releasing+fluoride varnish; GI-B (FR+CPP): fluoride-releasing+CPP-ACP; GII-A (NFR+FV): non-fluoride-releasing+fluoride varnish; G II-B (NFR+CPP): non-fluoride-releasing+CPP-ACP; CPP-ACP: casein phosphopeptide-amorphous calcium phosphate

Group (n = 10) each		Vickers microhardness unit (gm/mm^2^)					Mean rank	Test statistic
		Mean	SD	Min	Max	Median		
Control groups (adhesive only)	GI-C (FR)	199.03	5.82	193	206.33	198.83	15.85	χ2=45.43; df=5; p=<.001
	GII-C (NFR)	153.3	22.14	126	187	156	6.7	
Test groups (adhesive plus RM agent)	GI-A (FR+FV)	292.16	17.54	264.66	317.33	288.66	54.45	
	GI-B (FR+CPP)	300.23	62.54	210	378.66	289.83	24.35	
	GII-A (NFR+FV)	167.33	2.5	165	173	166.83	35.15	
	GII-B (NFR+CPP)	198.96	58.77	132.66	279	167	46.5	

**Table 3:** Pairwise comparison between two individual groups for shear bond strength (SBS) (Dunn-Bonferroni test). Test statistics: Dunn-Bonferroni test. * p<0.05 -statistically significant, the "Adj. p-value" is obtained by multiplying the p-value by the number of tests. GI-C (FR): only fluoride-releasing adhesive; GII-C (NFR): only non-fluoride-releasing adhesive; GI-A (FR+FV): fluoride-releasing+fluoride varnish; GI-B (FR+CPP): fluoride-releasing+CPP-ACP; GII-A (NFR+FV): non-fluoride-releasing+fluoride varnish; GII-B (NFR+CPP): non-fluoride-releasing+CPP-ACP; CPP-ACP: casein phosphopeptide-amorphous calcium phosphate

Inter-pair comparison between groups(n = 10)		Test statistc	Std. error	Std. test statistic	p-value	Adj. p
GI-C (FR)	GII-C (NFR)	9.15	7.8	1.17	0.241	1
GI-C (FR)	GI-A (FR+FV)	-38.6	7.8	-4.95	< .001*	< .001*
GI-C (FR)	GI-B (FR+CPP)	-8.5	7.8	-1.09	0.276	1
GI-C (FR)	GII-A (NFR+FV)	-19.3	7.8	-2.47	.013*	0.201
GI-C (FR)	GII-B (NFR+CPP)	-30.65	7.8	-3.93	< .001*	.001*
GII-C (NFR)	GI-A (FR+FV)	47.75	7.8	6.12	< .001*	< .001*
GII-C (NFR)	GI-B (FR+CPP)	17.65	7.8	2.26	.024*	0.356
GII-C (NFR)	GII-A (NFR+FV)	-28.45	7.8	-3.65	< .001*	.004*
GII-C (NFR)	GII-B (NFR+CPP)	-39.8	7.8	-5.1	< .001*	< .001*
GI-A (FR+FV)	GI-B (FR+CPP)	30.1	7.8	3.86	< .001*	.002*
GI-A (FR+FV)	GII-A (NFR+FV)	19.3	7.8	2.47	.013*	0.201
GI-A (FR+FV)	GII-B (NFR+CPP)	7.95	7.8	1.02	0.308	1
GI-B (FR+CPP)	GII-A (NFR+FV)	-10.8	7.8	-1.38	.166*	1
GI-B (FR+CPP)	GII-B (NFR+CPP)	-22.15	7.8	-2.84	.005*	0.068
GII-A (NFR+FV)	GII-B (NFR+CPP)	-11.35	7.8	-1.45	0.146	1

**Table 4:** Pairwise comparison between two individual groups for hardness (Dunn-Bonferroni test). Test statistics: Dunn-Bonferroni test. * p<0.05 -statistically significant, the "Adj. p-value" is obtained by multiplying the p-value by the number of tests. GI-C (FR): only fluoride-releasing adhesive; GII-C (NFR): only non-fluoride-releasing adhesive; GI-A (FR+FV): fluoride-releasing+fluoride varnish; GI-B (FR+CPP): fluoride-releasing+CPP-ACP; GII-A (NFR+FV): non-fluoride-releasing+fluoride varnish; GII-B (NFR+CPP): non-fluoride-releasing+CPP-ACP; CPP-ACP: casein phosphopeptide-amorphous calcium phosphate

Inter-pair comparison between groups (n = 10)		Test statistic	Std. error	Std. test statistic	P-value	Adj. p
GI-C (FR)	GII-C (NFR)	21.8	7.81	2.79	.005*	0.079
GI-C (FR)	GI-A (FR+FV)	-18.05	7.81	-2.31	.021*	0.312
GI-C (FR)	GI-B (FR+CPP)	-18.75	7.81	-2.4	.016*	0.245
GI-C (FR)	GII-A (NFR+FV)	13.5	7.81	1.73	0.084	1
GI-C (FR)	GII-B (NFR+CPP)	7.5	7.81	0.96	0.337	1
GI-A (FR+FV)	GI-B (FR+CPP)	-0.7	7.81	-0.09	0.929	1
GI-A (FR+FV)	GII-C (NFR)	39.85	7.81	5.1	< .001*	< .001*
GI-A (FR+FV)	GII-A (NFR+FV)	31.55	7.81	4.04	< .001*	.001*
GI-A (FR+FV)	GII-B (NFR+CPP)	25.55	7.81	3.27	.001*	.016*
GI-B (FR+CPP)	GII-C (NFR)	40.55	7.81	5.19	< .001*	< .001*
GI-B (FR+CPP)	GII-A (NFR+FV)	32.25	7.81	4.13	< .001*	.001*
GI-B (FR+CPP)	GII-B (NFR+CPP)	26.25	7.81	3.36	.001*	.012*
GII-C (NFR)	GII-A (NFR+FV)	-8.3	7.81	-1.06	0.288	1
GII-C (NFR)	GII-B (NFR+CPP)	-14.3	7.81	-1.83	0.067	1
GII-A (NFR+FV)	GII-B (NFR+CPP)	-6	7.81	-0.77	0.442	1

**Table 5:** Distribution of adhesive remnant index (ARI) scores within each group. RM: remineralizing agent; GI-C (FR): only fluoride-releasing adhesive; GII-C (NFR): only non-fluoride-releasing adhesive; GI-A (FR+FV): fluoride-releasing+fluoride varnish; GI-B (FR+CPP): fluoride-releasing+CPP-ACP; GII-A (NFR+FV): non-fluoride-releasing+fluoride varnish; GII-B (NFR+CPP): non-fluoride-releasing+CPP-ACP; CPP-ACP: casein phosphopeptide-amorphous calcium phosphate

Group (n = 10) each		ARI score categories				Total against rows	Median	Mean rank
		Score-0 n	Score-1 n	Score-2 n	Score-3 n			
Control groups (adhesive only)	GI-C (FR)	1	4	3	2	10	1.5	34
	GII-C ( NFR)	0	4	5	1	10	2	36.65
Test groups (adhesive plus RM agent)	GI-A (FR+FV)	1	5	3	1	10	1	30.6
	GI-B (FR+CPP)	3	3	2	2	10	1	28.35
	GII-A (NFR+FV)	0	6	3	1	10	1	32.35
	GII-B (NFR+CPP)	4	4	1	1	10	1	21.05
Total against columns		9	26	17	8	60		

**Table 6:** Comparison of frequency of distribution of adhesive remnant index (ARI) scores among different groups. Expected observations at least one cell has less than five observations, so the assumptions for the Chi-square test are not fulfilled. RM: remineralizing agent; GI-C (FR): only fluoride-releasing adhesive; GII-C (NFR): only non-fluoride-releasing adhesive; GI-A (FR+FV): fluoride-releasing+fluoride varnish; GI-B (FR+CPP): fluoride-releasing+CPP-ACP; GII-A (NFR+FV): non-fluoride-releasing+fluoride varnish; GII-B (NFR+CPP): non-fluoride-releasing+CPP-ACP; CPP-ACP: casein phosphopeptide-amorphous calcium phosphate

Group (n = 10) each		Groups- score index				Test static
		ARI score categories				χ2=14.35; df=5; p=.499; Cramér’s V = 0.28
		Score-0 n	Score-1 n	Score-2 n	Score-3 n	
Control groups (adhesive only)	GI-C (FR)	1.5	4.33	2.83	1.33	
	GII-C ( NFR)	1.5	4.33	2.83	1.33	
Test groups (adhesive plus RM agent)	GI-A (FR+FV)	1.5	4.33	2.83	1.33	
	GI-B (FR+CPP)	1.5	4.33	2.83	1.33	
	GII-A (NFR+FV)	1.5	4.33	2.83	1.33	
	GII-B (NFR+CPP)	1.5	4.33	2.83	1.33	

## Discussion

The term "white spot lesion" (WSL) denotes the initial visible sign of demineralization on the smooth surfaces of enamel. Despite significant advancements, one of the primary concerns that needs to be addressed by clinicians during fixed orthodontic appliance treatment is the prevention and management of WSL. Maintaining oral hygiene during fixed orthodontic therapy is difficult, leading to prolonged plaque accumulation on tooth surfaces and consequently resulting in the demineralization of enamel. The presence of brackets and other attachments hinders the effectiveness of natural self-cleaning mechanisms due to their uneven surfaces [2-4,11]. Thus, it is more important to modify the risk factors for dental caries and prioritize care strategies that prevent the onset of WSL and promote the remineralization of lesions, if any occur.

The enamel demineralization process commences with two discrete phases alternating with each other. The initial stage is characterized by subsurface lesions that demonstrate dissolution of the subsurface lesion of the enamel with an intact outer surface layer of the enamel. The fluorides are effective at this stage [5,6,11]. The next stage is surface relaxation, which is characterized by the loss of minerals from the outermost layer. During this stage, if the proper concentration of calcium and phosphate ions is maintained in the immediate environment of the enamel surface, remineralization ensues with the passage of these ions back to the enamel surface. This is where the remineralization agents play an important role [5,6,11]. The WSL is formed at the outer edge of the bracket, and these brackets are connected using orthodontic adhesives. Attempts were made to use fluoride-releasing orthodontic composites to address demineralization, but their effectiveness was not fully achieved [2]. This in vitro study aimed to investigate if the addition of remineralization agents to composite materials enhances the resistance of enamel surfaces to demineralization.

We established an artificial demineralized region surrounding the bracket surface that was attached to the tooth surface. We next examined the impact of coating with remineralizing agents on the surface hardness when used in conjunction with both fluoride and non-fluoride adhesives. The Vickers microhardness test (VMT) offers the advantage of being simple, straightforward, easy to perform, and non-intrusive. It possesses the capacity to employ the identical specimen multiple times, thereby reducing the likelihood of experimental errors [12]. This test is based on the principle that a well-remineralized enamel surface has higher hardness values (HV). We also explored whether the coating of a remineralizing agent has an adverse effect on the shear bond strength of the given adhesive. We also evaluated the effect of the remineralizing agent on the type of bond failure, as this may affect the enamel surface during debonding procedures.

**Figure 6:**
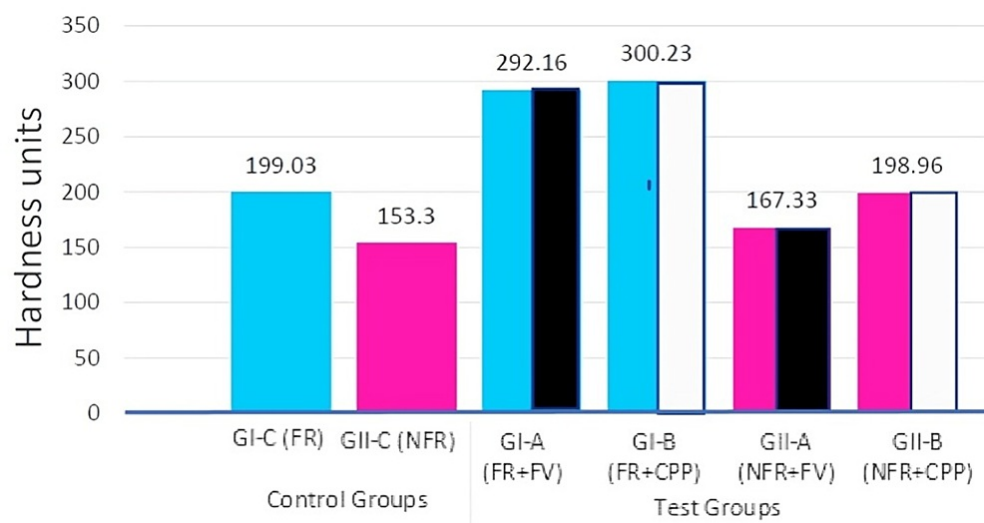
Graphic representation of the hardness value (HV) of the groups. GI-C (FR): only fluoride-releasing adhesive; GII-C (NFR): only non-fluoride-releasing adhesive; GI-A (FR+FV): fluoride-releasing+fluoride varnish; GI-B (FR+CPP): fluoride-releasing+CPP-ACP; GII-A (NFR+FV): non-fluoride-releasing+fluoride varnish; GII-B (NFR+CPP): non-fluoride-releasing+CPP-ACP

The present investigation provides convincing evidence that the use of a remineralizing agent with composites significantly enhances the hardness of the enamel (Figure 6). This effect is particularly noticeable when using a fluoride-releasing adhesive in combination with either kind of agent (Table 2, Table 4). The FR adhesive in combination with CPP-APP (GI-B 300.23 + 62.54) yields the greatest values. The HV of this group is the highest and significantly higher than that of all groups tested with NFR adhesive (P< 001). Nevertheless, there is no observed statistical significance in relation to GI-A (FR+FV), and the average difference between these two groups is merely 7 units. When used in combination with both composite adhesives, adding CPP-ACP (300.23 + 62.54, 198.6 + 58.77) has a greater positive additive impact than the FV agent (292.16 + 17.54, 167.33 + 2.5).

The findings of our investigation align with the previous research conducted by Trinajstic [13], which determined that the remineralizing capacity of fluoride-releasing adhesive was twice as great as that of non-fluoride-releasing composite. Our investigation found that the FR adhesive, when combined with both agents (FR+FV-292.16 + 17.54, FR+CPP-300.23 + 62.54), has a remineralizing capability that is 1.5 to 1.7 times greater than NFR combinations (NFR+FV-167.33 + 2.5, NFR+CPP-198.6 + 58.77). A study by Shivananda [14] supported the use of fluoride varnish, while another investigation [15] found that CPP-ACP had a greater capacity for remineralization compared to fluoride varnish. The variations may have occurred because of differences in the concentration and type of fluorides used in the respective studies.

We also investigated whether the inclusion of remineralizing agents had a negative impact on bond strength. Nevertheless, there is a clear positive correlation between the SBS and all the combinations as compared to the control group. Table 1 and Table 3 indicate that GI-A exhibits the greatest enhancement in bond strength, with a value of 11.59 + 0.5 MPa. There is an observed increase in the bond strength, which falls within the range of 1 to 3 MPA. However, this has no practical importance, as the bond strength required to endure the stresses exerted during treatment falls within the range of 5.9 to 7.8 MPa [16].

In our study, we did not find a specific pattern in the SBS results for the combinations tested. The NFR with CPP and the FR with FV exhibited higher bond strengths. The bond strengths documented in our investigation are comparatively lower than those reported in earlier studies. Previous studies found, in general, that FR adhesives have a higher SBS compared to NFR adhesives [17-21]. The literature is inconclusive regarding the bond strength of composites when combined with different remineralizing agents [17,18]. A study by Anis Tabrizi [19] found that adding CPP-ACP to adhesive makes the bond stronger compared to using 5% sodium fluoride varnish. However, Çehreli [20] reported that adding CPP-ACP with Transbond Plus (FR) exhibited greater bond strength than adding CPP-ACP with Transbond XT (NFR). Another study reported that the addition of CPP-ACP to non-fluoride-releasing adhesive increased the bond strength of the bracket more than the addition of fluoride varnish [21].

The present study also evaluated the pattern of bond failure in all the groups by ARI scores [10]. This index quantifies the enamel surface loss on the tooth following the debonding of brackets after the completion of the treatment. We measured the ARI objectively by overlaying a calibrated graph on the stereographic images of the bracket bases. The score-2 category showed the highest frequency of bond failure across all groups, followed by the score-1 category, indicating that about 50% of the adhesive bonds to the enamel (Table 5). The interface between the bracket and adhesive is the failure site, offering the most advantageous location for safe debonding due to a reduced risk of enamel fracture. The only noticeable finding is that maximum bond failures with ARI-0 occurred at the interface between enamel and the adhesive interface with CPP (GI-B and GII-B)-coated brackets, regardless of the type of adhesive used. This type of score is associated with less adhesive on the teeth, thereby reducing the time and providing less chance for enamel loss during the adhesive removal. In the present study overall, there is no statistically significant difference in the type of bond failure between the different groups (Table 6).

The ARI scores of the current study are in concordance with previous studies, which have shown that most of the failure rates with FR adhesives occur at the bracket/adhesive interface [17,18]. Another study also found that there is no significant difference in the frequency of bond failures between FR and NFR adhesives [22]. In contrast to the present study, the ARI score-0 was most repeated in fluoride varnish-coated brackets compared to CPP-ACP when bonded with NFR adhesive.

There are few clinical studies available in the literature that evaluate the effectiveness of remineralizing agents. One such study [23] concluded that CPP-ACP is very effective in reducing demineralized enamel lesions during orthodontic treatment. A comparative study by Rúbia [24] demonstrated similar results with two different fluoride varnishes (Fluorphat® and Duraphat®) in increasing the remineralization of active WSL. The remineralization potential of CPP-ACP-based cream and fluoride varnish over molar incisor hypomineralization was found to be equally effective in achieving demineralization [25].

The region surrounding the orthodontic brackets presents challenges in terms of cleanliness and is thus susceptible to caries attacks. The initial lesion is rapid and very active. It is better to prevent it before the lesion is established. Keeping that in mind, we undertook the present investigation with the objective of averting the development of a white spot lesion, and if the occurrence of the lesion was unavoidable, it should be reversed in order to prevent further progress. In an attempt to solve this problem, we combined fluoride varnish and CPP-ACP with a fluoride-releasing adhesive. We have identified a significant advantage in combining CPP-ACP with fluoride-releasing adhesive. In clinical applications, the orthodontist can apply CPP-ACP around the brackets after bonding and advise the patient to do so for 28 days. CPP-ACP is commercially available as GC Tooth Mousse. The study recommends using CPP-ACP for at least 28 days to achieve its effectiveness.

This study was done under controlled conditions in a research laboratory. Hence, confounding patient factors could not be considered. The current study does not examine the remineralization potential, bond strength, or bond failure over a range of time periods. The teeth that were taken for this study have no blood or nerve supply, which may differ from the natural teeth that are in the oral cavity. Demineralization of the enamel was not evaluated at the outset of this investigation.

## Conclusions

The adhesive that releases fluoride has a larger potential for remineralization than the adhesive that does not release fluoride. Additionally, the use of fluoride varnish, or CPP-ACP, improves the remineralization capability of the fluoride-releasing adhesive. Adding CPP-ACP to fluoride-releasing adhesive demonstrated a higher level of surface hardness than adding fluoride varnish.
